# Green beards in the light of indirect genetic effects

**DOI:** 10.1002/ece3.5484

**Published:** 2019-08-02

**Authors:** Barbora Trubenová, Reinmar Hager

**Affiliations:** ^1^ Institute of Science and Technology Austria Klosterneuburg Austria; ^2^ Evolution and Genomic Systems, School of Biological Sciences, Manchester Academic Health Science Center, Faculty of Biology, Medicine and Health The University of Manchester Manchester UK; ^3^ Present address: Computational and Evolutionary Biology, Faculty of Life Sciences Michael Smith Building Manchester UK

**Keywords:** altruism, green beards, indirect genetic effects

## Abstract

The green‐beard effect is one proposed mechanism predicted to underpin the evolution of altruistic behavior. It relies on the recognition and the selective help of altruists to each other in order to promote and sustain altruistic behavior. However, this mechanism has often been dismissed as unlikely or uncommon, as it is assumed that both the signaling trait and altruistic trait need to be encoded by the same gene or through tightly linked genes. Here, we use models of indirect genetic effects (IGEs) to find the minimum correlation between the signaling and altruistic trait required for the evolution of the latter. We show that this correlation threshold depends on the strength of the interaction (influence of the green beard on the expression of the altruistic trait), as well as the costs and benefits of the altruistic behavior. We further show that this correlation does not necessarily have to be high and support our analytical results by simulations.

## 1 INTRODUCTION

One of the explanations for the evolution of altruism is the so‐called green‐beard mechanism (Dawkins, 1976; Hamilton, 1964). In this concept, a single gene or several tightly linked genes encoding altruistic behavior need to meet three requirements: (a) cause its bearer to behave altruistically, (b) display an observable and distinctive trait (the "green beard"), and (c) recognize the signal and modify the behavior accordingly (Dawkins, 1976; Keller & Ross, 1998; Queller, 2008). An allele causing altruism has the capacity to recognize individuals with copies of itself, helping the individuals that carry them, and thus helping to propagate itself (Dawkins, 1976; Grafen, 1998). Therefore, the altruistic behavior of an individual is just a result of the selfish behavior of the green‐beard gene (Dawkins, 1976).

A problem with the green‐beard effect is that any mutant alleles that produce the advertising trait without providing the helping behavior—a cheater—will cause its bearer to have higher fitness and will, therefore, be selected for (Gardner & West, 2010). To avoid this, it is assumed that both the signaling and the altruistic trait must be encoded by the same gene or a set of very tightly linked genes. Therefore, so‐called "green beards" are predicted to be rare.

Despite the initial skepticism about whether such situations occur in nature, green‐beard‐like genes have been documented in different species. Keller and Ross (1998) discovered a green‐beard gene in the red fire ant, *Solenopsis invicta*, where a linked set of alleles causes workers to kill homozygous queens that lack the green‐beard allele, while not killing individuals that contain it. Workers appear to distinguish between carriers and noncarriers by a transferable odor cue (Grafen, 1998; Keller & Ross, 1998). The "green beard" is a chemical carried on the queens' cuticle. Keller and Ross (1998) showed that all components of a green‐beard effect are present—a detectable phenotypic feature, the ability to recognize the feature, and differential responses toward individuals with and without the feature. Moreover, all these features are mediated by a group of closely linked genes.

Another example of green‐beard scenario comes from yeast (Smukalla et al., 2008). Flocculation is a formation of flocs of flakes of yeast that helps to protect them from the damage from chemicals, for instance, alcohol. Flocculation is caused by a protein, a product of the gene FLO1. Cells that make this protein have to pay a cost: They grow more slowly than cells that do not express it. However, only cells expressing FLO1 can stay inside of the floc. Even though some of these cells on the outer side of the floc die, those inside the floc survive and pass the altruistic gene to the next generation. Cells without the FLO1 gene do not form flocs and die if exposed to harsh chemicals.

While several modelling approaches have been developed to study the evolution of altruism (mainly based on game theory), we find that a quantitative genetics framework based on indirect genetic effects is ideally suited to model the green‐beard scenario. Indirect genetic effect models are based on the premise that trait expression is influenced not only by an individual's own genes (direct genetic effects – DGEs) but also by genes expressed in social partners—indirect genetic effects (IGEs) (Agrawal, Brodie, & Wade, 2001; Cheverud, 1984; Dickerson, 1947; Moore, Brodie, & Wolf, 1997; Wolf, 2000; Wolf, Brodie, Cheverud, Moore, & Wade, 1998; Wolf, Brodie, & Moore, 1999). They have received significant interest in the past few years, especially in the context of the evolution of social behaviors (Akçay & Van Cleve, 2012; Bijma, 2010b; Bijma, Muir, Ellen, Wolf, & Arendonk, 2007a; Bijma Muir & Arendonk, 2007b; Hadfield & Thomson, 2017; McGlothlin & Brodie, 2009; McGlothlin, Moore, Wolf, & Brodie, 2010; McGlothlin, Wolf, Brodie, & Moore, 2014; Muir, 2005; Trubenová & Hager, 2012). One of the major advantages of IGE models is that they are based on parameters that can be readily determined (Bijma, 2010a; McGlothlin & Brodie, 2009). Here, we show that these models are ideally suited to the analysis of green‐beard effects because the signaling–recognition mechanism in green beards is, by definition, captured by IGEs. The IGE models thus allow easy formalization of the green‐beard scenario based on measurable parameters, such as the strength of selection and interaction strength (Bijma, 2010a; McGlothlin & Brodie, 2009) and allow to express benefit and cost for the interactants in these terms.

McGlothlin et al. (2010) briefly investigated the green‐beard scenario using IGE models and showed the conditions for altruism to evolve, in different scenarios: (a) when individuals assort *randomly* with respect to the signaling trait (a badge), and there is *no genetic correlation* between the traits, and when they assort *nonrandomly*, (b) *with* or (c) *without the genetic correlation* between the signaling and the altruistic trait. As the authors pointed out, the evolution of badge‐based altruism is unlikely without any genetic covariance between the behavior and the signaling traits. Here, we build on their model and complement their results by investigating the fourth scenario: when individuals assort randomly (no relatedness), but there is a genetic correlation between the traits. We use an IGE model to specifically focus on the correlation between the signaling and altruistic trait required for the evolution of the latter. We argue that altruistic traits can evolve and persist even in random populations of unrelated individuals if the correlation between the signaling and altruistic trait is above a given threshold. We show that this correlation threshold depends on the strength of the interaction (influence of the green beard on the expression of the altruistic trait), as well as the strength of social and nonsocial selection and support our analytical results with agent‐based simulations.

## 2 IGE MODEL

To analyze the sufficient correlation between traits in the green‐beard scenario, we will use the multivariate model describing the phenotype of the focal individual (McGlothlin et al., 2010)

(1) $$z_{i}=a_{i}+e+\left(N-1\right){\Psi z}^{\prime },$$

where **z_i_** and **a_i_** are column vectors describing phenotypes and genotypes (additive genetic values) of the focal individual, respectively, **e** is a vector of residual values (environmental influences), and **z′** denotes the mean phenotype of the focal individual's social partners in a group of size *N*. The square matrix Ψ describes IGEs. Note that IGEs can be affected by interactions among individuals in a nonlinear way, described in (Trubenová & Hager, 2012), and capture epistasis.

**Figure 1:**
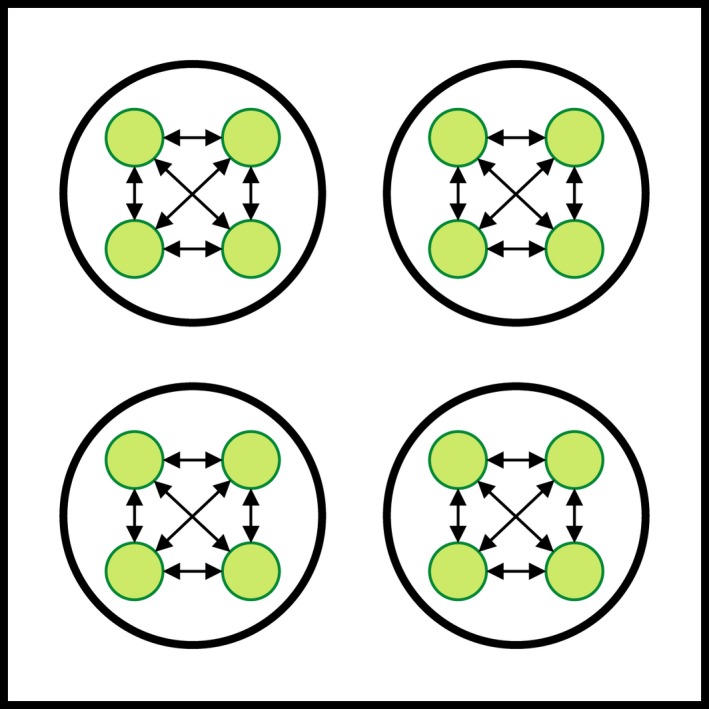
Scheme of the model. Individuals (green circles) interact (arrows) with each other within the group (black circles), but not between groups. Groups are of the same size and population (black square) size is constant

We assume that the fitness of the focal individual is not only a function of its own phenotype, but is also affected by the phenotypes of others. The effect of an individual's own phenotype on its own fitness is described by a nonsocial selection gradient *β* **_N_** (Lande, 1979; Lande & Arnold, 1983; Moore et al., 1997), while the effect of interactant phenotypes on the focal individual's fitness is captured by a social selection gradient *β* **_S_** (Agrawal, 2001; Bijma & Wade, 2008; McGlothlin et al., 2010; Queller, 1992; Westneat, 2012; Wolf et al., 1999). Both selection gradients are column vectors with each element quantifying the fitness effect of a corresponding phenotypic trait. We further assume that groups are of the same size and the individuals do not interact between groups (Figure 1).

Following previous IGE models (Bijma & Wade, 2008; McGlothlin et al., 2010; Wolf et al., 1999), we define individual fitness as

(2) $$W_{i}=\alpha +z_{j}^{T}\beta_{N}+\left(N-1\right)z^{\prime T}\beta_{S}$$

where the column vector *β* **_N_** is a nonsocial selection gradient describing the effect of an individual's traits on its own fitness, *β* **_S_** is a social selection gradient describing the effects of other traits on the fitness of the focal individual (Lande & Arnold, 1983; Frank, 1997; Wolf et al., 1999; McGlothlin et al., 2010, and *α* is a positive constant.

The genotypic response to selection $\Delta \bar{\bar{a}}$ is defined as the difference between the mean genotypic value of offspring and the mean genotypic value of the parental generation. To determine the genetic response to selection, we adapted the expression derived by (McGlothlin et al., 2010, equation 18) for a case of a population with equally sized, nonoverlapping groups of *N* individuals. The authors derived an expression for the phenotypic response to selection (change between the mean phenotypic value of offspring and the mean phenotypic value of the parental generation) as $\Delta \bar{\bar{z}}=\left[{\left[I-\left(N-1\right)\Psi \right]}^{-1}\right]\Delta \bar{\bar{a}}$. Thus, omitting the factor ${\left[I-(N-1)\Psi \right]}^{-1}$ leads to the genotypic response to selection

(3) $$\begin{array}{c} \Delta \bar{\bar{a}}=G\left[I-\left(N-2\right)\Psi^{T}+r\left(N-1\right)\Psi^{T}\right]{\left[I-\left(N-2\right)\Psi^{T}-\left(N-1\right)\Psi^{T}\Psi^{T}\right]}^{-1}\beta_{N}\\ +\left(N-1\right)G\left(rI+\Psi^{T}\right){\left[I-\left(N-2\right)\Psi^{T}-\left(N-1\right)\Psi^{T}\Psi^{T}\right]}^{-1}\beta_{s} \end{array}$$

where *N* is the group size, *r* is relatedness between individuals, and **G** is an additive variance—covariance matrix (Lande, 1979).

## 3 ANALYTICAL RESULTS

We apply the above‐described IGE model to the green‐beard scenario. We consider two traits of interest: a signaling trait and an altruistic trait (*z_s_* and *z_a_*, respectively), encoded by their corresponding genes (genotype **a **= [*a*_s_, *a*_a_]^T^). We further assume that the genotypic values are normally distributed around 0. The presence of a green beard (a signalling trait) positively enhances the expression of altruism in social partners, which can be captured in matrix form as $\Psi =\left[\begin{array}{cc} 0 & 0\\ \Psi & 0 \end{array}\right]$ where Ψ>0. The altruistic behavior (phenotype) of each individual is given by the genotypic value of its altruistic trait (or, rather, "predisposition to altruistic behavior") mediated by the signaling of its social partners (Equation 1).

While the signaling trait has no direct influence on the fitness of any individual, the altruistic trait increases the direct fitness of others (social selection) and decreases the fitness of its bearer (nonsocial selection). Thus, we can write social selection and nonsocial selection gradients as *β* **_S_** = [0, *β_S_*]*^T^* and *β* **_N_** = [0, *β_N_*]*^T^*, respectively, where *β_S_* > 0 and *β_N_* < 0. The altruistic behavior is not directed at a specific individual—all social partners experience the same behavior from a particular individual.

As in randomly formed groups, the relatedness between interacting individuals is expected to be *r* = 0, the response to selection (Equation 3) is simplified to

(4) $$\begin{array}{c} \Delta \bar{\bar{a}}=G\left[I-\left(N-2\right)\Psi^{T}\right]{\left[I-\left(N-2\right)\Psi^{T}-\left(N-1\right)\Psi^{T}\Psi^{T}\right]}^{-1}\beta_{N}\\ +\left(N-1\right){G\Psi }^{T}{\left[I-\left(N-2\right)\Psi^{T}-\left(N-1\right)\Psi^{T}\Psi^{T}\right]}^{-1}\beta_{s}. \end{array}$$

Filling in Ψ, *β* **_S_**, and *β* **_S_** for our specific scenario allows further simplification (Appendix A) and leads to

(5) $$\begin{array}{c} =\left[\begin{array}{c} G_{11},G_{12}\\ G_{21},G_{22} \end{array}\right]\left[\begin{array}{c} 0\\ \beta_{N} \end{array}\right]+\left(N-1\right)\left[\begin{array}{c} G_{11},G_{12}\\ G_{21},G_{22} \end{array}\right]\left[\begin{array}{c} 0,\Psi \\ 0,0 \end{array}\right]\left[\begin{array}{c} 0\\ \beta_{N} \end{array}\right]\\ =\left[\begin{array}{c} \left(N-1\right)\beta_{S}\Psi G_{11}+\beta_{N}G_{21}\\ \left(N-1\right)\beta_{S}\Psi G_{12}+\beta_{N}G_{22} \end{array}\right]\\ =G\beta_{N}+\left(N-1\right){G\Psi }^{T}\beta_{S} \end{array}$$

where *G*_11_ = var(*a_s_*) is the genotypic variance of the signaling trait, *G*_22_ = var(*a_a_*) is the genotypic variance of the altruistic trait, and *G*_12_ = G_21_ is the covariance between the two. For the altruistic trait to evolve, the response to selection of this trait must be positive, yielding the condition

(6) $$(\left(N-1\right)\beta_{S}\Psi G_{12}+\beta_{N}G_{22})>0.$$

As the correlation coefficient is defined as $\rho =\frac{G_{12}}{\sqrt{G_{11}G_{22}}}$, we can express a threshold correlation coefficient between signaling and altruistic traits necessary for the latter to evolve

(7) $$\rho_{T}=\frac{-\beta_{N}}{\left(N-1\right)\beta_{S}\Psi }\sqrt{\frac{\text{var}(a_{a})}{\text{var}(a_{s})}}.$$

Note that as *β_N_* has a negative sign, the threshold correlation coefficient is positive if Ψ is positive. If Ψ is negative, the correlation must be negative, and below the threshold. However, altruism can still evolve.

**Figure 2:**
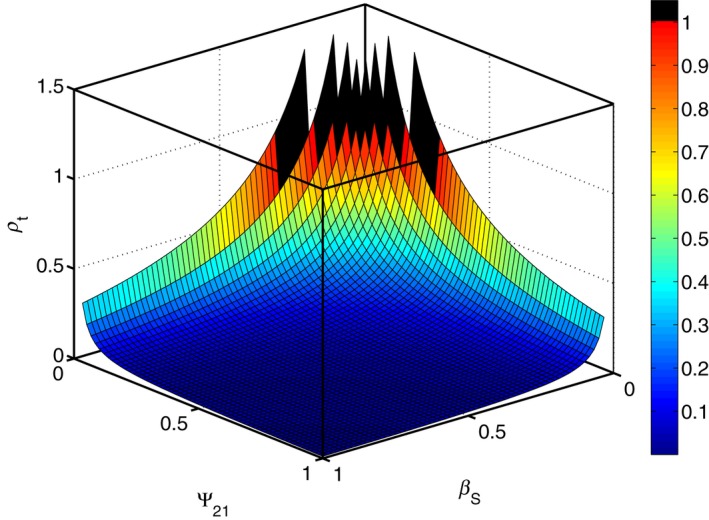
Threshold correlation between altruistic and signaling traits. The correlation ρ necessary for the evolution of altruism in green beards depends on both interaction strength Ψ and the social selection gradient *β*. The stronger the interaction or social selection gradient, the lower the necessary correlation between the signaling and altruistic trait. However, when these are too low, the altruistic trait cannot evolve as the required threshold is higher than the maximum possible correlation. *β_N_* = −1, genetic variances of both traits are equal

Figure 2 shows the dependence of the correlation between traits necessary for the evolution of altruism, for different interaction strength Ψ and social selection strength *β_S_*. If this threshold is higher than 1, it means that the altruistic behavior cannot evolve in this particular circumstances (parameter space).

## 4 SIMULATION RESULTS

We support our analytical results discussed in the main manuscript with agent‐based simulations carried out in MATLAB and Python.

We ran agent‐based simulations of *mN* individuals randomly assorted into *m* groups consisting of *N* individuals each. Initial genotypic values of both the signaling and the altruistic genes are drawn from the bivariate normal distribution with mean of 0, standard deviation of 1/3, and specified correlation between the two. The altruistic behavior (phenotype) of each individual is given by the genotypic value of its altruistic gene (predisposition to altruistic behavior), as well as by the level of signaling of its social partners (Equation 1). The fitness of each individual is calculated using Equation 2. The population size remains constant, and the individuals contributing to the next generation are selected randomly with probability proportional to their fitness. We assume asexual populations with no recombination and no new mutations. The new generation of individuals is reshuffled and randomly assigned to groups.

### 4.1 Response to selection

**Figure 3:**
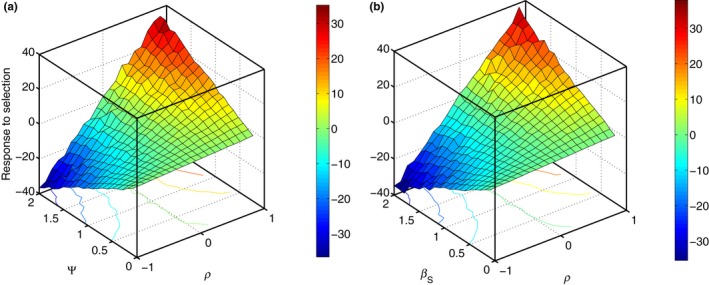
Response to selection in green beards. Dependence of the response to selection on social selection gradient *β_S_*, interaction strength Ψ, and correlation ρ between the signaling and the altruistic trait in green beards. Response to selection at each point is calculated as a mean of 200 simulations of 50 individuals in five groups. Simulations were carried out in MATLAB

Equation 4 shows that the response to selection of both traits depends linearly on indirect genetic effects Ψ as well as on the strength of selection (both social and nonsocial) and the correlations between both the traits. This analytical result is supported by simulations, as illustrated in Figure 3.

**Figure 4:**
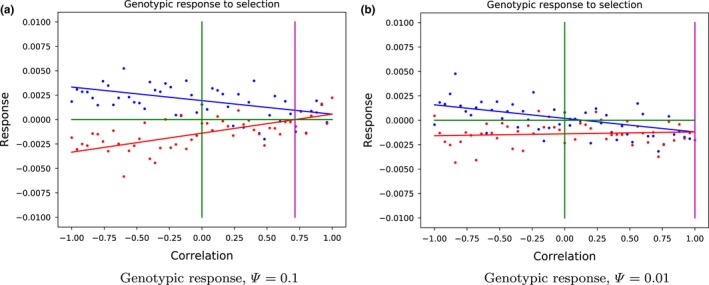
Response to selection depends on the correlation between altruistic and signaling traits. Comparison of analytical solution and simulations, when *β_N_* = −1 and *β_S_* = 1. Dots are results of simulations, mean of 100 trials. Lines represent analytical predictions. Blue dots and lines represent response to selection of the signaling trait, while the response of the altruistic trait is shown in red. Green lines represent axes. The purple lines show the analytically calculated minimal correlation between the traits required for the altruistic trait to evolve

**Figure B1:**
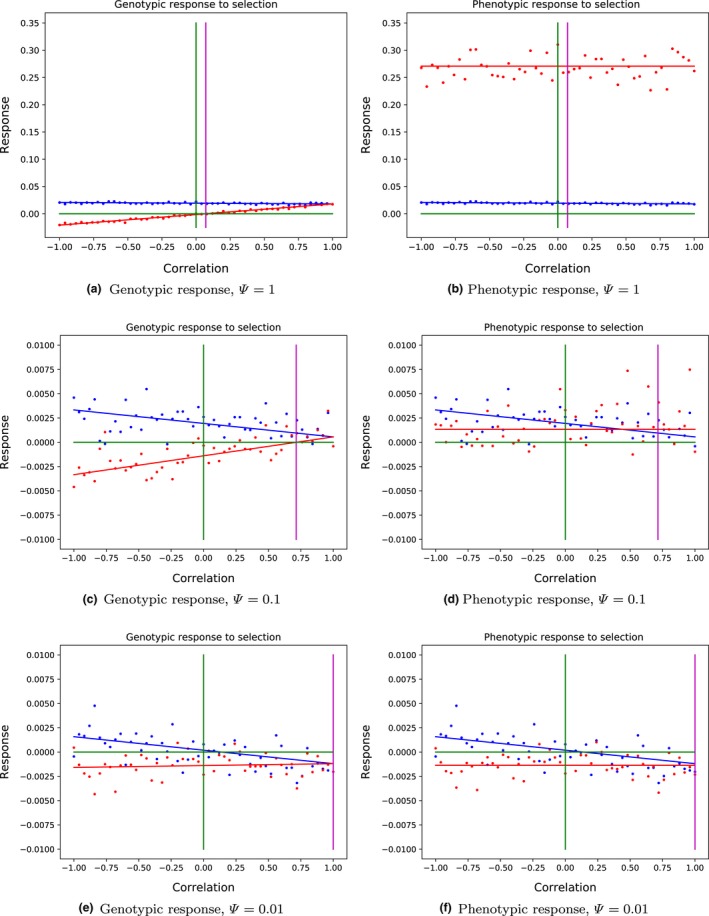
Response to selection depends on the correlation between altruistic and signaling traits. Comparison of analytical solution and simulations, when *β_N_* = −1 and *β_S_* = 1. Dots are results of simulations; lines are analytical predictions. Blue dots and lines represent the response to selection of the signaling trait, while the response of the altruistic trait is shown in red. Green lines represent axes. The purple lines show the analytically calculated minimal correlation between the traits required for the altruistic trait to evolve. Means are calculated from 100 independent trials

To support our analytical derivation of the minimal correlation necessary for increasing the genotypic value of the altruistic gene, we simulated a population of 50 groups consisting of 15 interacting individuals each, for a range of correlation values between the signaling and the altruistic trait. The response to selection is calculated as the difference between the mean genotypic values of the parental and the offspring generations. The mean response to selection for each parameter set was calculated from 100 trials. The results of the simulations displayed in Figure 4 show that if the correlation is below the required threshold, the mean response to selection is negative, while if the correlation is above the threshold, the response is positive, as predicted by our theoretical results. This means that the mean genetic value of the altruistic gene (predisposition to altruistic behavior) has increased in the offspring generation. See Appendix B, Figure B1 for additional simulations of different parameter sets.

### 4.2 Long‐term evolution

**Figure 5:**
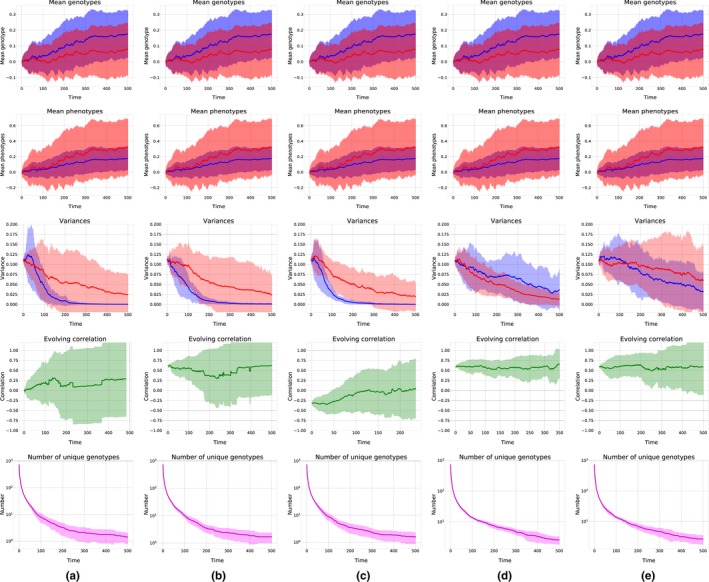
Long‐term evolution for different parameter sets. Blue represents the signaling trait, while the altruistic trait is shown in red. Lines represent means and shaded areas standard deviation. All simulations: *β_S_* = 1, *β_N_* = −1, *N* = 15, *m* = 50. Column a: ρ = .0, Ψ = 1. Column b: ρ = .6, Ψ = 1. Column c: ρ = −.3, Ψ = 1. Column d: ρ = .6, Ψ = .01. Column e: ρ = .6, Ψ = .1. Means are calculated from 20 independent long‐term trials

**Figure B2:**
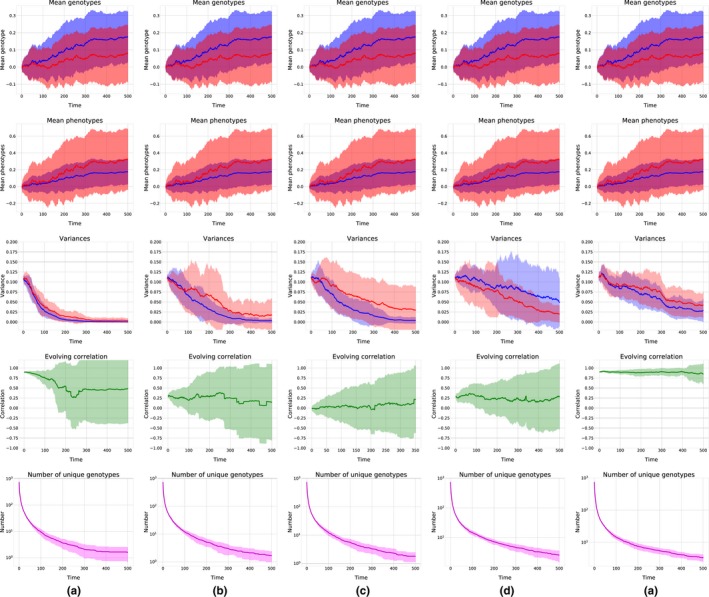
Long‐term evolution for different parameter sets. Blue represents the signaling trait, while the altruistic trait is shown in red. Lines represent means and shaded areas standard deviation. All simulations: *β_S_* = 1, *β_N_* = −1, *N* = 15, *m* = 50. Column a: ρ = .9, Ψ = 1. Column b: ρ = .3, Ψ = .3. Column c: ρ = 0, Ψ = .3. Column d: ρ = .3, Ψ = .01. Column e: ρ = .9, Ψ = .1. Means are calculated from 20 independent long‐term trials

To simulate long‐term evolution, we simulated evolving populations for 500 generations, for various *m* (number of groups) and *N* (group size). As expected, after some time, fixation occurred, and genetic variance was lost from the population. Figure 5 shows the simulation results: evolving mean genotypic and phenotypic values of both traits, decreasing genetic variances, and correlation between the traits and the number of unique haplotypes for various *m* and *N* (average of 20 trials for each parameter set). We observed that even in cases when the mean genotypic value of the altruistic trait decreased, the overall expression of altruistic behavior increased due to the increased signaling genotypic values. See Appendix B, Figure B2 for additional simulations of different parameter sets.

**Figure 6:**
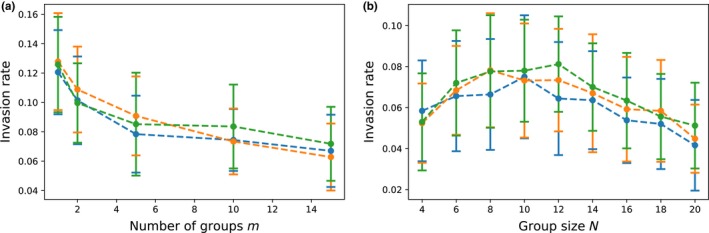
Invasion rate depends on the group, as well as population size. With increasing number of groups (fixed *N* = 10), cheaters invasion rate was decreasing. However, this trend was not obvious for the increasing group size (fixed *m* = 10), where the invasion rate peaked at *N* = 10. Blue line–initial correlation ρ = 0, red line ρ = .45 and blue line ρ = .9. Means and standard deviation calculated from 50 repeats with 200 independent trials for each parameter set

To investigate the stability of the evolved altruistic population, we let the population evolve for 500 generation and then introduced a single individual with a high genetic value of the signaling, but a low value of altruistic trait—a cheater. We simulated the population for another 500 generations and recorded whether the cheater haplotype invaded the population or was eradicated. We varied the group size *N*, number of groups *m*, and the initial correlation ρ between the signaling and the altruistic trait. Invasion rate is calculated from 200 independent trials, and mean invasion rate and standard deviation are calculated from 50 repeats of 200 evolution trials for each parameter set. Figure 6 shows the results of the simulations. We observed that while the initial correlation between the traits did not have any observable impact, the rate of invasions decreased with increasing number of groups. This could be expected, as the fixation probability of an allele decreases with the overall population size *mN*. However, this trend was not so clear with increasing group size, as the invasion rate slightly increased at the beginning.

## 5 DISCUSSION

Most population genetics, as well as game theoretic models analyzing the evolution of altruism assume a binary division of phenotypes (and genotypes as well): altruists and cheaters. While undoubtedly helpful, the biological reality surely differs—most complex and social traits, such as altruistic behavior, are not binary but expressed on continuous scales and the environment likely mediates their expression. IGE models are naturally set up to deal with such situations, offering more realistic insight into many aspects of social behavior. Several social interactions can be formalized and analyzed by these models as easily as the green‐beard scenario. Recently, models of social evolution with continuous traits have been developed Mullon, Keller, and Lehmann (2016), but these are still rare. Even rarer are the models of social evolution that consider multiple traits, as we did in our model. Below, we discuss the main finding of our analysis.

It should be noted, that our approach to study green‐beard scenario is limited by the assumption of mediated, but nondiscriminate altruism toward an individual's social partners. Each individual's level of altruism is determined by its group, and all social partners experience the same benefit from a given individual. However, the same individual would behave differently in a different group. Therefore, it is rather a "group's green beard" than an "individual's green beard" that plays a role, and thus, it could be argued that our model is not the green‐beard model as originally suggested by Dawkins. One possibility to use IGEs while tending to this problem is to consider sequential pairwise interactions between individuals, rather than a simultaneous interaction (J. McGlothlin, personal communication).

### 5.1 Correlation threshold

McGlothlin et al. (2010) derived conditions for altruism to evolve in three different scenarios. First, the authors showed that in a case when individuals assort to groups randomly (i.e., the relatedness is zero) and there is no genetic correlation between the signaling and the altruistic trait; the strength of interaction Ψ would have to be very strong in comparison with the strength of nonsocial selection. Then, the authors assumed that individuals assort nonrandomly with respect to the signaling trait, with or without the genetic correlation, which lessened the condition on the strength of interaction. In this manuscript, we complement their results, and we explicitly assume that groups are formed randomly (the relatedness is zero) and show that altruism can still evolve, even if both interaction strength Ψ and selection strength *β_S_* are of the same magnitude.

Using IGE models, we were able to determine the necessary correlation between the signaling and the altruistic trait for the latter to evolve (Equation 7). We show how this threshold depends both on interaction strength and selection gradients (Figure 2). This correlation threshold increases with the increasing cost associated with the altruistic behavior (negative nonsocial selection gradient), and decreases with the interaction strength, as well as with the positive social selection experienced by the recipients of the altruistic behavior. However, for small values of both interaction strength Ψ and selection strength *β_S_* (relative to negative nonsocial selection) the response to selection is always negative, even if both traits are completely correlated (essentially encoded by the same locus).

While we are not aware of any other study investigating the correlation between the signaling and the altruistic trait in the green‐beard scenario, Kirkpatrick and Barton (1997) obtained very similar results when investigating the evolution of female mating preference. The authors derived an expression for the genetic correlation between the male's signaling trait and female preference. While the authors did not use an IGE framework, the expression they derived closely matches Equation 7. This is not surprising, as both situations bear strong similarities: both the green beard and the male signaling trait do not directly contribute to an individual's fitness, but enhance traits in others (altruistic behavior or the willingness to mate) that have direct fitness consequences.

### 5.2 Response to selection

Equation 4 offers more profound insight into the forces shaping the evolution of altruistic behavior and the signaling trait. The second term of the response to selection of the altruistic trait *β_N_* var(*a_a_*) describes the direct adverse effect of an altruistic trait on the fitness of its carrier. However, if the correlation between the signaling and altruistic trait is strong enough, it may be compensated by the indirect response to selection, described by the first term $\left(N-1\right)\beta_{S}\Psi \text{cov}\left(a_{a}a_{s}\right)$.

The first term $\left(N-1\right)\beta_{S}\Psi \text{var}\left(a_{s}\right)$ in Equation 4 shows that the interaction Ψ induces a positive response to selection in the signaling trait—the high values of signaling genes are rewarded through eliciting altruistic behaviors from other, leading to positive correlation between genotypic values and fitness. As McGlothlin et al. (2010) pointed out, both traits will tend to run away together. However, this effect is partially opposed by the second term *β_N_*cov(*a_a_a_s_*), describing the indirect response to selection, which has an adverse fitness effect due to the correlation with altruistic behavior. Thus, while the correlation between traits enhances the evolution of an altruistic trait, it slows down the evolution of the signaling trait. Because IGEs are associated with the altruistic trait and not the genetic correlation between the two, the signaling trait evolves in a positive direction.

Our agent‐based simulations support the analytical results for the correlation threshold. While the response to selection in a particular population greatly varies, the mean response closely matched the predicted values for given correlations. For correlations above the predicted threshold, the mean response of the altruistic trait is positive, meaning that the offspring's mean genotypic value for altruistic behavior has increased. The mean genotypic value of the signaling trait also increased, for any value of correlation. However, the response decreased with the increasing correlation, as suggested by Equation 4. As above, this is because the higher values of the signaling trait are associated with the higher values of the costly altruistic trait.

The manifestation of the altruistic trait (phenotype) depends highly on the population structure and the level of signaling. Interestingly, we observed that even for some parameter sets, where the mean genotypic value of the altruistic trait evolved toward negative values, the mean phenotypic value of altruistic behavior was positive and increasing. This was caused by the evolution of the signaling trait—higher signaling levels increased altruistic behavior, even though the genotypic values evolved in the opposite direction. This points to the importance of distinguishing between the genotypic response to selection derived here, and the phenotypic response to selection derived by McGlothlin et al. (2010). The phenomenon of opposite responses to selection was recently investigated by Fisher and Pruitt (2019). Among other reasons, the authors concluded that they typically occur when social selection opposes direct selection, as happens in our case.

In our model, the expression of the altruistic trait depends on the overall (absolute) level of signaling, meaning that more social partners would elicit more altruistic behavior from the focal individual. In such a case, whether the individual behaves altruistically or not depends on the size of the group it belongs to. However, it would also be reasonable to assume that the altruistic response depends on the mean, rather than the absolute level of signaling in the group. In such a case, group size would not affect the expression of the trait, and we would not observe the discrepancy between the signs of the phenotypic and genotypic response to selection. In other words, if the mean, not the absolute level of signaling determines the expression of the altruistic trait, the phenotypic and genotypic response to selection should be in the same direction (Appendix B). The most realistic model would be somewhere in between these two extremes: The level of signaling would be partially "diluted" by the increasing number of social partners. However, introducing such "dilution coefficient" would add complexity to our model. We leave the investigation of how the level of dilution would affect the correlation threshold for further studies.

### 5.3 Long‐term evolution

A positive response to selection in one generation does not guarantee the long‐term increase in the level of altruism, as the correlation between the traits will also develop with the evolution of the traits. Our simulations of long‐term evolution suggest that the response to selection is positive for a number of generations if the initial correlation is above the calculated threshold (Equation 7). The mean genotypic values of both traits increase, as does the overall expression of the altruistic behavior. After a while, the increase flattens up and reaches a stable level. The genetic variance of both traits decreases, as does the correlation between both traits. This is not surprising—as we consider no new mutations and no recombination, we soon run out of variance, with only one haplotype present in the population. Incorporating diploidy, sexual reproduction, and recombination into the model would likely lead to more restrictive conditions for the evolution of altruism.

Interestingly, mean phenotypic values are increasing even for correlations below the threshold, and even when the mean genotypic value is negative, the mean phenotypic value can be positive. This is because the expression of the trait is strongly determined by Ψ and driven by the evolution of the signaling trait.

Jansen and van Baalen (2006) investigated a more general case of a green‐beard scenario—where not only green, but multiple different beard colors exist, with altruistic individuals helping only those with the same beard color (beard chromodynamics). The authors observed that strong coupling between the signaling trait and the altruistic beard leads to fixation of a single colour and eventual crash of the altruistic population when cheaters emerge. On the other hand, when the coupling was loose, the fixation did not occur—instead, several groups of different colors coexisted. When cheaters of the same colour invaded a population of altruists, the population of a particular colour declined and was taken over by another group of altruistic individuals. Jansen and van Baalen (2006) suggest these cycles could repeat indefinitely, stabilizing in boom‐bust dynamics.

In our model, there is no binary division of phenotypes between altruists and cheaters, and the genotypic values of both traits are continuous and mediated by the phenotypes of social partners. Furthermore, altruistic behavior is mediated by the phenotypes of social partners but is nondiscriminatory—every social partner experiences the same benefit from a particular individual. However, "cheaters" can be simulated as individuals that have high genetic values for the signaling trait, but low genetic values of the altruistic trait.

While the Janssen and Goldstone (2006) model is different from ours, some of the conclusions can be compared between the models. In our model, the strong correlation between the traits also leads to quick fixation. However, we did not observe any effect of the initial correlation on the probability of cheater invasion. This is not surprising, as, after 500 generations, only one haplotype was left in the population in every trial. Furthermore, we observed that the increasing number of groups had a negative impact on the invasion rate. This is also to be expected, as the probability of fixation of a new mutation is inversely proportional to the size of the population. However, increasing the group size first lead to an increased invasion rate, then to a decreased rate. The peak of the invasion rate was observed when the number of individuals within one group was the same as the number of groups. This might be caused by the increased benefits that the cheater receives in bigger groups, partially compensating for the decreasing fixation probability.

The utility of the IGE models has been highlighted numerous times (Bijma & Aanen, 2010; Bijma, Muir, Ellen, et al., 2007a; Bijma, Muir, & Van Arendonk, 2007b; Hadfield & Thomson, 2017; McGlothlin et al., 2010, 2014; Nonacs & Hager, 2010; Wilson, 2013). In this study, we used IGE models to shed new light on the notorious problem of green‐beard scenarios. We have shown that even if the green beard and the altruistic behavior are not encoded by the same gene, altruistic behavior can evolve if the correlation between the genotypic values of both traits is sufficiently high.

We complemented our analytical results with agent‐based simulations and confirmed that even in the long term, altruistic behavior can evolve though the green‐beard mechanism and persist in the population. Furthermore, our simulations show that such altruistic populations can be reasonably resistant to the invasion of cheaters. However, we did not assume any recombination between the traits, which could increase the probability of cheaters invading. This, as well as other possible extensions including variable population sizes and mutation events, still need to be investigated.

## CONFLICT OF INTEREST

None declared.

Data archival location: There are no data to be archived.

## AUTHOR CONTRIBUTIONS

BT: analyzed the model and wrote the article; RH: supervised the analysis and wrote the article.

## Data Availability

Simulation code and additional figures available from Github: https://github.com/Trubenova/Green-Beard.git

## APPENDIX A DERIVATION OF THE ANALYTICAL RESULTS IN THE GREEN‐BEARD SCENARIO

We consider two traits of interest: a signaling and an altruistic trait (*z_s_* and *z_a_*, respectively), encoded by their corresponding genes (*a_s_* and *a_a_*, respectively). The presence of a green beard (a signaling trait) positively alters the expression of altruism in social partners, which can be captured in matrix form as $\Psi =\left[\begin{array}{cc} 0 & 0\\ \Psi & 0 \end{array}\right]$ where Ψ> 0. The altruistic trait increases the direct fitness of others but decreases the fitness of its bearer, while the signaling trait has no direct influence on the fitness of any individual: *β* **_S_** = [0, *β*_S_] and *β* **_N_** = [0, *β_N_*], respectively, where *β_S_* > 0 and *β_N_* < 0.

In randomly formed groups, relatedness between interacting individuals is *r* = 0. Thus, using Equation 3, we can express the response to selection:

(A1) $$\begin{array}{c} \Delta \bar{\bar{a}}=G\left[I-\left(N-2\right)\Psi^{T}\right]{\left[I-\left(N-2\right)\Psi^{T}-\left(N-1\right)\Psi^{T}\Psi^{T}\right]}^{-1}\beta_{N}\\ +\left(N-1\right){G\Psi }^{T}{\left[I-\left(N-2\right)\Psi^{T}-\left(N-1\right)\Psi^{T}\Psi^{T}\right]}^{-1}\beta_{s}. \end{array}$$

Filling in Ψ, *β* **_S_** and *β* **_S_** for the green‐beard scenario leads to

$$\begin{array}{c} =\left[\begin{array}{c} G_{11},G_{12}\\ G_{21},G_{22} \end{array}\right]\left[\begin{array}{c} 1,-(N-2)\Psi \\ 0,1 \end{array}\right]{\left[\begin{array}{c} 1,-(N-2)\Psi \\ 0,1 \end{array}\right]}^{-1}\left[\begin{array}{c} 0\\ \beta_{N} \end{array}\right]\\ +\left(N-1\right)\left[\begin{array}{c} G_{11},G_{12}\\ G_{21},G_{22} \end{array}\right]\left[\begin{array}{c} 0,\Psi \\ 0,0 \end{array}\right]\left[\begin{array}{c} 1,-(N-2)\Psi \\ 0,1 \end{array}\right]\left[\begin{array}{c} 0\\ \beta_{N} \end{array}\right] \end{array}$$

as ${\Psi \Psi }^{T}$ gives a zero matrix. This further simplifies to

(A2) $$\begin{array}{c} =\left[\begin{array}{c} G_{11},G_{12}\\ G_{21},G_{22} \end{array}\right]\left[\begin{array}{c} 0\\ \beta_{N} \end{array}\right]+\left(n-1\right)\left[\begin{array}{c} G_{11},G_{12}\\ G_{21},G_{22} \end{array}\right]\left[\begin{array}{c} 0,\Psi \\ 0,0 \end{array}\right]\left[\begin{array}{c} 0\\ \beta_{N} \end{array}\right]\\ +\left[\begin{array}{c} \left(N-1\right)\beta_{S}\Psi G_{11}+\beta_{N}G_{21}\\ \left(N-1\right)\beta_{S}\Psi G_{12}+\beta_{N}G_{22} \end{array}\right] \end{array}$$

that can also be expressed as (but only for these parameters)

(A3) $$=G\beta_{N}+\left(N-1\right){G\Psi }^{T}\beta_{S}$$

where *G*_11_ = var (*a_s_*) is the genotypic variance of the signaling trait, *G*_22_ = var (*a_a_*) is the genotypic variance of the altruistic trait and *G*_12_ = *G*_21_ is the covariance between the two.

## APPENDIX B ADDITIONAL SIMULATIONS

We support our analytical results discussed in the main manuscript with agent‐based simulations carried out in MATLAB or Python.

Each trial begins by generating a population consisting of *m* groups of *N* individuals. Genotypic values of both the signaling and the altruistic trait are drawn from the normal distribution with mean set to 0 and standard deviation to 1/3. Individual phenotypes and fitness are calculated according to the genotypes (and phenotypes) of their social partners. The fitness of each individual is calculated using Equation 2. A new generation is created from the old one by randomly drawing *MN* individuals from the old generation with the probability proportional to their fitness. The new generation of individuals is reshuffled and randomly assigned to groups.

To support our analytical derivation of the minimal correlation necessary for the altruistic trait to evolve, we calculated the mean response to selection for a range of correlation values as an average of 100 independent trials for each parameter set (each data point). The response to selection in each trial was calculated as the difference between the mean genotypic value of the offspring and the parental generation. The simulations were carried out in Python. Figure B1 shows the results of simulations for various parameter sets.

Figure B2 shows additional simulations of long‐term evolution for various parameter sets. We simulated 20 trials for each parameter set.
